# Tripterygium wilfordii Hook F accelerates CD4^+^ T-cell recovery in ART-treated people living with HIV with incomplete immune reconstitution: a longitudinal cohort study

**DOI:** 10.3389/fphar.2026.1854636

**Published:** 2026-06-12

**Authors:** Liyuan Zheng, Fada Wang, Xiaojing Song, Leidan Zhang, Yanling Li, Wei Lv, Yang Han, Wei Cao, Taisheng Li

**Affiliations:** 1 Department of Infectious Diseases, Peking Union Medical College Hospital, Peking Union Medical College and Chinese Academy of Medical Sciences, Beijing, China; 2 State Key Laboratory for Complex, Severe, and Rare Diseases, Peking Union Medical College Hospital, Beijing, China; 3 Center for AIDS Research, Chinese Academy of Medical Sciences, Beijing, China; 4 Peking Union Medical College Hospital, Beijing, China; 5 Chinese Academy of Medical Sciences and Peking Union Medical College, Beijing, China

**Keywords:** antiretroviral therapy, CD4 T-cell recovery, HIV, immune activation, incomplete immune reconstitution, inflammatory chemokines, Tripterygium wilfordii Hook F

## Abstract

**Background:**

Incomplete immune reconstitution (INR) affects 9%–45% of ART-treated people living with HIV (PLWH) and is associated with increased morbidity and mortality. Chronic immune activation and inflammatory signaling, particularly via the IP-10/CXCL10 pathway, are central to its pathogenesis. Whether Tripterygium wilfordii Hook F (TwHF), an immunomodulatory agent with established anti-inflammatory properties, can improve CD4^+^ T-cell recovery in virologically suppressed PLWH with INR remains unclear.

**Methods:**

We conducted a retrospective longitudinal cohort study at Peking Union Medical College Hospital. ART-treated, virologically suppressed PLWH with persistent CD4^+^ T-cell counts <350 cells/μL were enrolled and classified into a TwHF group (n = 32, 10 mg three times daily) or a matched control group (n = 31). Participants were followed at five predefined time points spanning 12 months pre-treatment through 12 months post-discontinuation. Peripheral blood immunophenotyping assessed CD4^+^ T-cell subsets (naïve and memory), CD4/CD8 ratio, and CD8^+^ T-cell activation markers. A cytokine substudy measured IP-10/CXCL10 and eotaxin in 20 TwHF-treated and 14 control participants using multiplex immunoassay. Linear mixed-effects models were used for longitudinal analysis.

**Results:**

TwHF was associated with a significantly accelerated rate of CD4^+^ T-cell recovery compared with controls (group × time interaction coefficient 4.98, P < 0.001), with median counts of 254 vs. 222 cells/μL at 12 months (P = 0.011). This gain was attributable predominantly to memory CD4^+^ T-cell expansion (216 vs. 164 cells/μL, P < 0.001), while naïve CD4^+^ T-cell counts remained unchanged. The CD4/CD8 ratio improved more rapidly in the TwHF group (0.409 vs. 0.278 at month 12, P = 0.008). A clinically meaningful response (≥50 cells/μL/year) was achieved in 75.0% of TwHF-treated vs. 9.7% of controls. Within-person IP-10/CXCL10 levels declined significantly after TwHF treatment (median Δ −31.96 pg/mL, P = 0.017), particularly in good immunological responders. Immunological gains attenuated after treatment discontinuation, suggesting a treatment-dependent effect. Routine hematological and renal parameters remained within normal limits throughout follow-up, with no significant between-group differences. No serious adverse events were observed.

**Conclusion:**

TwHF accelerates CD4^+^ T-cell reconstitution in virologically suppressed PLWH with INR, primarily through memory subset expansion and modulation of IP-10/CXCL10 inflammatory signaling, supporting its potential as an adjunctive immunomodulatory strategy.

## Introduction

Despite the widespread use of antiretroviral therapy (ART) and sustained virologic suppression, a substantial proportion of people living with HIV (PLWH) fail to achieve adequate immune recovery (Acquired Immunodeficiency Syndrome Professional Group, Society of Infectious Diseases, Chinese Medical Association, Chinese Center for Disease Control and Prevention 2024; Yang et al., 2020; Zhang and Ruan, 2023; Battegay et al., 2006). This condition, commonly referred to as incomplete immune reconstitution (INR), is characterized by persistently low CD4^+^ T-cell counts despite long-term viral control and affects approximately 9%–45% of treated individuals (Yang et al., 2020). INR has been consistently associated with an increased risk of AIDS-related and non-AIDS-related morbidity and mortality, underscoring the need for effective strategies to promote immune reconstitution beyond viral suppression alone (Zhang and Ruan, 2023; Yan et al., 2023; G et al., 2012; Pacheco et al., 2015).

Accumulating evidence indicates that chronic immune activation and systemic inflammation play a central role in the pathogenesis of INR (Yan et al., 2023; G et al., 2012; Rajasuriar et al., 2010). Even in the setting of durable viral suppression, persistent inflammatory signaling—particularly along interferon-γ–associated chemokine pathways—has been linked to impaired CD4^+^ T-cell recovery (Metzemaekers et al., 2017). Elevated levels of inflammatory mediators such as interferon-γ–induced protein 10 (IP-10/CXCL10) are consistently associated with poor immunological outcomes, reflecting a dysregulated immune milieu that limits T-cell survival and homeostasis (Rajasuriar et al., 2010; Kroeze et al., 2021; Ploquin et al., 2016; Wan et al., 2023). Despite extensive investigation, therapeutic interventions targeting these inflammatory processes have yielded limited and inconsistent benefits in INR populations (Zhang and Ruan, 2023; G et al., 2012).

Tripterygium wilfordii Hook F (TwHF) is a standardized botanical preparation with established immunomodulatory properties that is distinct from isolated triptolide derivatives. At the mechanistic level, triptolide—the principal bioactive constituent of TwHF—suppresses NF-κB activation and downstream transcription of pro-inflammatory mediators including TNF-α, IL-1β, and IL-6, while concurrently inhibiting the JAK/STAT1 signaling axis to attenuate IFN-γ–induced chemokine expression, including CXCL10 (Shan et al., 2023; Liu, 2011; Shi et al., 2023; He et al., 2024). These converging actions position TwHF as a candidate modulator of the chronic interferon-γ–driven inflammatory milieu that characterizes INR. Our group previously demonstrated, through combined multi-omics and network pharmacology analyses of clinical samples from TwHF-treated INR patients, that TwHF exerts immunomodulatory effects via coordinated suppression of T-cell activation pathways (Liu et al., 2022). Separately, our group conducted a randomized, placebo-controlled phase II trial evaluating (5R)-5-hydroxytriptolide (LLDT-8)—a synthetic triptolide analogue and a chemically distinct compound from the TwHF botanical preparation—which demonstrated enhanced CD4^+^ T-cell recovery and reduced inflammatory markers including IP-10 in virologically suppressed PLWH with INR (ClinicalTrials.gov NCT04084444) (Cao et al., 2023). However, these studies primarily focused on changes in total CD4^+^ T-cell counts and systemic inflammatory markers, and did not delineate the contribution of specific CD4^+^ T-cell subsets or characterize the longitudinal dynamics of immune reconstitution, including the durability of immunological effects following treatment withdrawal.

To address these unresolved questions, we conducted a single-center longitudinal cohort study to evaluate the immunological effects of TwHF in ART-treated, virologically suppressed PLWH with INR. By integrating longitudinal analyses of CD4^+^ T-cell recovery, CD4^+^ T-cell subset composition, and inflammatory cytokine dynamics before, during, and after treatment, we aimed to characterize the cellular basis and temporal nature of immune reconstitution associated with TwHF exposure. Our study provides real-world evidence on how immunomodulatory intervention influences immune recovery in INR, with particular emphasis on memory CD4^+^ T-cell dynamics and pathway-specific inflammatory signaling.

## Materials and methods

### Study design and population

This retrospective longitudinal cohort study was conducted within the AIDS outpatient follow-up cohort at Peking Union Medical College Hospital. Adult patients (≥18 years) with confirmed HIV-1 infection were eligible if they had received continuous ART for at least 4 years and maintained sustained virologic suppression (HIV-1 RNA <20 copies/mL) for a minimum of 3.5 years (Acquired Immunodeficiency Syndrome Professional Group, Society of Infectious Diseases, Chinese Medical Association, Chinese Center for Disease Control and Prevention, 2024).

INR was defined as a persistent CD4^+^ T-cell count <350 cells/μL on at least two consecutive measurements obtained ≥3 months apart during the period of virologic suppression, despite long-term viral suppression. Patients meeting these criteria were included and classified into the TwHF group or the control group according to whether TwHF had been prescribed during routine clinical follow-up. TwHF pills (Zhejiang Deende Pharmaceutical Co., Ltd., China) were administered orally at a standard dosage of 10 mg three times daily as part of routine clinical care, based on prior evidence of efficacy in this indication (Liu et al., 2022; Cao et al., 2023). The present study is a retrospective analysis of prospectively collected clinical data from these patients; all clinical decisions, including TwHF prescription, were made by the treating physician independent of this research. Written informed consent for data use was obtained from all participants (see Ethical approval). Safety monitoring included routine hematologic assessments as well as liver and renal function tests at scheduled follow-up visits.

**FIGURE 1:**
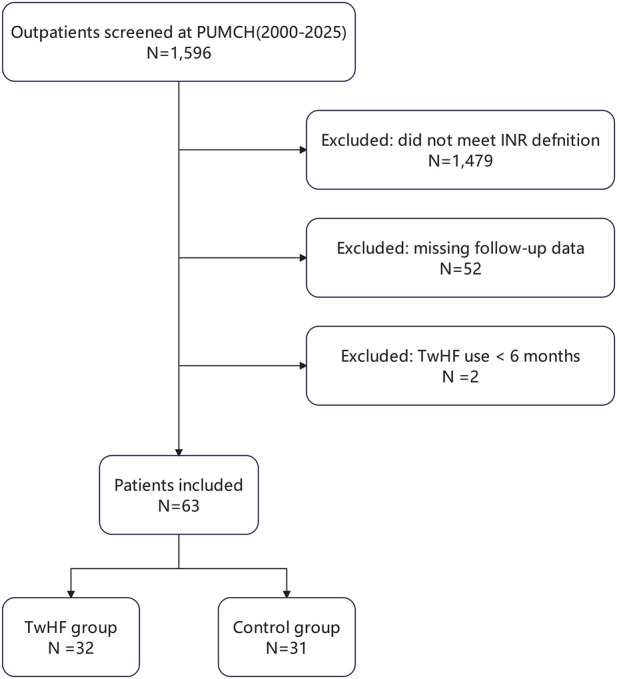
Study flow diagram. Flow diagram of participant screening, inclusion, and follow-up. A total of 1,596 PLWH were screened; 63 ART-treated, virologically suppressed individuals with INR were included.

Medication adherence was monitored at each scheduled follow-up visit (approximately every 3 months) through structured patient self-report. At each visit, participants were asked to quantify missed doses since the preceding visit. Participants who reported more than five missed doses within any 3-month interval received adherence counseling; those who met this threshold at two consecutive follow-up visits were withdrawn from the study. Participants who reported more than ten missed doses within any 3-month period were immediately withdrawn. Participants excluded for adherence-related reasons are reflected in the study flow diagram (Figure 1).

For clarity, two baseline time points were defined: immunological parameters prior to ART initiation were used to characterize disease severity at treatment onset, whereas all longitudinal analyses were referenced to cohort entry following sustained viral suppression and prior to TwHF initiation or matched follow-up.

## Ethical approval

The study was carried out in accordance with the principles of Good Clinical Practice and the Declaration of Helsinki of 1975. The study protocol for this retrospective analysis of clinical data was approved by the institutional review board of Peking Union Medical College Hospital (1-23Pj1189). All participants provided written informed consent for the use of their clinical data for research purposes. TwHF was prescribed as part of routine clinical practice; the present study involves retrospective analysis of data collected in the course of that clinical care and does not constitute a clinical trial.

### Inclusion and exclusion criteria

Inclusion criteria: (1) age ≥18 years; (2) confirmed HIV-1 infection; (3) ART for ≥4 years; (4) sustained virologic suppression for ≥3.5 years; (5) CD4^+^ T-cell counts persistently <350 cells/μL on at least two consecutive measurements ≥3 months apart.

Exclusion criteria: (1) TwHF treatment duration <6 months; (2) missing immunological data at predefined time points; (3) presence of opportunistic infections, autoimmune diseases, malignancies, or systemic immunosuppressive therapy during the observation period; (4) active hepatitis B or C virus coinfection requiring antiviral therapy, active tuberculosis, or other clinically significant coinfections at the time of enrollment.

### Follow-up and study time points

Participants were followed at five predefined time points: Pre-M12 (12 months before TwHF initiation), TwHF-M0 (treatment start), TwHF-M6 (6 months of treatment), TwHF-M12 (12 months of treatment), and Post-M12 (12 months after treatment discontinuation).

### Immunophenotyping and lymphocyte subset analysis

Peripheral blood immunophenotyping was performed using three-color flow cytometry (Epics XL; Beckman Coulter, USA). Commercially available monoclonal antibody panels for CD3/CD4/CD8, CD3/CD16CD56/CD19, CD38/HLA-DR/CD8, CD28/CD8/CD4, and CD62L/CD45RA/CD4 were used with appropriate isotype controls (Dako, France). Absolute lymphocyte subset counts were calculated by integrating flow cytometry percentages with total leukocyte and lymphocyte counts. Naïve CD4^+^ T cells were defined as CD45RA^+^CD62L^+^, and memory CD4^+^ T cells as CD45RA^−^ cells.

### Inflammatory cytokine assessment

A predefined cytokine substudy was conducted using paired plasma samples from 20 TwHF-treated individuals (before and after treatment) and a single cross-sectional sample from 14 randomly selected control participants. For the cytokine substudy, TwHF-treated participants were stratified by CD4^+^ T-cell gain into upper and lower halves, and 10 individuals were randomly selected from each stratum (good responders: upper half; poor responders: lower half). Cytokine concentrations were measured using a multiplex bead-based immunoassay per manufacturer’s instructions. IP-10/CXCL10 and eotaxin were prespecified as primary cytokines of interest (Lv et al., 2022). Other analytes were considered exploratory.

### Outcome definitions

The primary immunological outcome was longitudinal change in CD4^+^ T-cell counts. Secondary outcomes included memory and naïve CD4^+^ T-cell subset composition, CD4/CD8 ratio, CD8^+^ T-cell activation markers (CD38^+^ and HLA-DR^+^), and inflammatory cytokine levels. A clinically meaningful immunological response was defined as a CD4^+^ T-cell increase of ≥50 cells/μL per year.

### Statistical analysis

Continuous variables were expressed as mean ± SD or median (IQR). Between-group comparisons used Mann–Whitney U tests or independent t-tests; within-group comparisons used Wilcoxon signed-rank tests. Longitudinal trajectories were analyzed using linear mixed-effects models with group, time, and group × time interaction as fixed effects. Categorical variables used χ^2^ or Fisher’s exact tests. All tests were two-sided (*P* < 0.05 significant). Analyses used SPSS v25.0 and GraphPad Prism v8.0.1.

## Results

### Baseline characteristics and safety

A total of 63 ART-treated, virologically suppressed PLWH with INR were included: 32 received TwHF and 31 served as controls (Figure 1). Groups were comparable in demographic characteristics and pre-ART baseline immunological parameters (Table 1). Of note, CD8^+^ T-cell counts at the Pre-M12 and TwHF-M0 time points were significantly lower in the TwHF group than in controls (550 vs. 839 cells/μL, *P* = 0.005; and 626 vs. 739 cells/μL, *P* = 0.044, respectively); this difference was not present at the pre-ART baseline (540 vs. 627 cells/μL, *P* = 0.611) and resolved during the treatment period (Supplementary Table S1). The pre-treatment CD8^+^ T-cell imbalance is addressed further in the Limitations section.

**TABLE 1:** Pre-ART baseline characteristics of study participants.

Variable	TwHF (n = 32)	Control (n = 31)	P value
Demographic and clinical characteristics			
Age (years)	41.3 ± 11.7	37.8 ± 8.3	0.179
Sex, female, n (%)	5 (15.6%)	7 (22.6%)	0.536
ART regimen (INSTI-based), n (%)	1 (4.0%)	3 (12.0%)	0.611
Time from ART to TwHF start (months)	42.9 ± 25.5	—	—
TwHF treatment duration (months)	12.2 ± 2.0	—	—
Pre-ART baseline immunological parameters			
CD4^+^ T cells (cells/μL)	63.0 ± 71.8	61.9 ± 44.3	0.394
CD4^+^ T cells (%)	6.2 ± 6.8	6.9 ± 8.5	0.523
CD8^+^ T cells (cells/μL)	631.6 ± 362.0	665.5 ± 334.0	0.611
CD8^+^ T cells (%)	59.3 ± 12.2	59.3 ± 12.9	0.989
CD4/CD8 ratio	0.119 ± 0.171	0.145 ± 0.258	0.768
Naïve CD4^+^ T cells (%)	13.2 ± 11.9	12.4 ± 13.6	0.445
Memory CD4^+^ T cells (%)	86.8 ± 11.9	87.6 ± 13.6	0.445
CD8^+^CD38^+^ (%)	78.6 ± 16.9	79.1 ± 18.4	0.704
CD8^+^HLA-DR^+^ (%)	58.8 ± 21.6	49.4 ± 20.2	0.080
HIV-1 RNA log_10_ (copies/mL) [n = 25, 20]	4.47 ± 1.06	4.45 ± 1.27	0.961

Values are mean ± SD or n (%). Between-group comparisons: independent t-tests, Mann–Whitney U tests, or Fisher’s exact tests. ART, antiretroviral therapy; INSTI, integrase strand transfer inhibitor; PLWH, people living with HIV.

Virologic suppression was maintained throughout the observation period in all participants. No participant experienced virologic blips (defined as a transient HIV-1 RNA between 20 and 200 copies/mL) or confirmed virologic failure (HIV-1 RNA >200 copies/mL on two consecutive measurements) during follow-up.

Routine hematological and biochemical safety monitoring data are presented in Supplementary Table S6. Mild, asymptomatic elevations in liver transaminases (≤2× upper limit of normal) were observed in eight participants (25.0%) in the TwHF group and three participants (9.7%) in the control group; all resolved spontaneously without dose modification. Routine hematological parameters—including white blood cell count (TwHF: 5.0 [4.1–6.3] × 10^9^/L at TwHF-M12; control: 5.9 [4.8–6.7] × 10^9^/L), hemoglobin (148.0 [135.8–152.8] vs. 149.0 [141.0–155.5] g/L), and platelet count (210.5 [163.5–256.2] vs. 218.0 [202.0–232.0] × 10^3^/μL)—remained within normal limits throughout the observation period, with no significant between-group differences at any time point (all *P* > 0.05; Supplementary Table S6). Serum creatinine was stable in both groups (TwHF: 74.0 [65.8–80.5] μmol/L; control: 75.5 [68.0–85.0] μmol/L at TwHF-M12; *P* = 0.604). No serious adverse events or treatment discontinuations due to adverse events were observed.

### CD4^+^ T-cell recovery and subset composition

**FIGURE 2:**
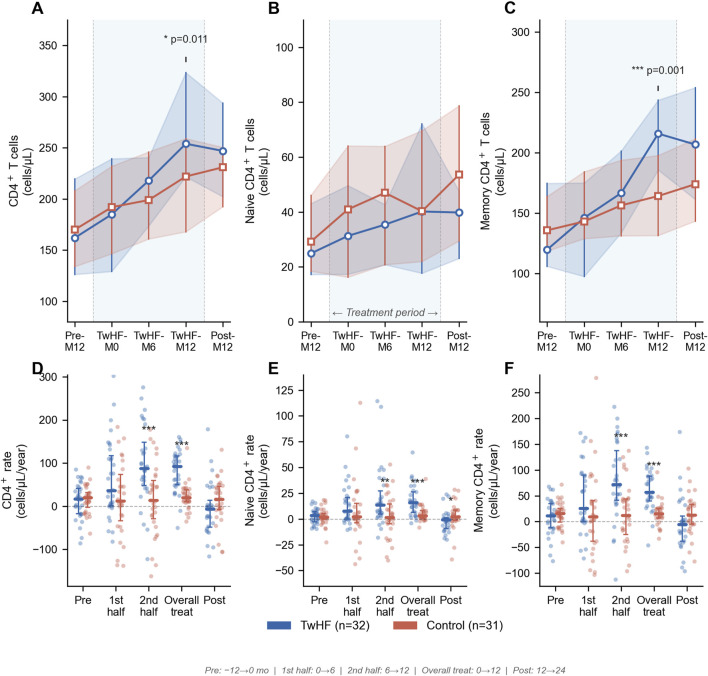
Longitudinal dynamics of CD4^+^ T-cell recovery and subset composition. **(A–C)** Absolute counts of total CD4^+^ T cells **(A)**, naïve CD4^+^ T cells **(B)**, and memory CD4^+^ T cells **(C)** at five predefined time points (Pre-M12, TwHF-M0, TwHF-M6, TwHF-M12, Post-M12). Data are medians with IQR (shaded area); the treatment period is highlighted. **(D–F)** Rates of change in total CD4^+^ **(D)**, naïve CD4^+^ **(E)**, and memory CD4^+^ **(F)** T-cell counts (cells/μL/year, annualised) during pre-treatment (Pre: −12→0 months), first half (0→6 months), second half (6→12 months), overall treatment (0→12 months), and post-treatment (12→24 months) periods. Horizontal lines = group medians; vertical bars = IQR; individual data points overlaid. *P < 0.05, **P < 0.01, ***P < 0.001 by Mann–Whitney U test.

During the intervention period, TwHF was associated with a significantly accelerated increase in CD4^+^ T-cell counts compared with controls (Figure 2). At TwHF-M12, median CD4^+^ T-cell counts were significantly higher in the TwHF group (254 vs. 222 cells/μL, *P* = 0.011). Linear mixed-effects modeling confirmed a significant group × time interaction (coefficient 4.98, *P* < 0.001; Supplementary Table S2), indicating a faster rate of CD4^+^ T-cell increase in the TwHF group that was most pronounced in the second half of treatment. Following treatment discontinuation, the between-group difference diminished progressively, consistent with a reversible, treatment-dependent effect.

Memory CD4^+^ T-cell counts increased more prominently in the TwHF group, reaching a significant between-group difference at TwHF-M12 (216 vs. 164 cells/μL, *P* < 0.001; Supplementary Table S3). In contrast, naïve CD4^+^ T-cell counts did not differ significantly between groups at any time point (Figures 2B,E; Supplementary Table S3), supporting preferential expansion of memory rather than naïve CD4^+^ T-cell subsets.

### CD4/CD8 ratio and CD8^+^ T-cell parameters

To confirm that the pre-treatment CD8^+^ T-cell imbalance did not confound the primary outcome, a sensitivity analysis was performed by including CD8^+^ T-cell counts at TwHF-M0 as a covariate in the linear mixed-effects model for CD4^+^ T-cell recovery. The group × time interaction coefficient remained unchanged (4.98, *P* < 0.001) after adjustment, confirming that the CD8^+^ baseline discrepancy did not influence the estimated treatment effect (Supplementary Table S5).

The CD4/CD8 ratio increased more rapidly in the TwHF group during the treatment period (group × time interaction *P* = 0.001; Supplementary Tables S2, S4). At TwHF-M12, the median CD4/CD8 ratio was 0.409 in the TwHF group versus 0.278 in controls (*P* = 0.008). By contrast, CD8^+^ T-cell counts did not differ significantly between groups during the treatment period at the cross-sectional level (TwHF-M6 through Post-M12, all *P* > 0.05; Supplementary Table S1), although linear mixed-effects modeling showed a nominally significant group × time interaction for CD8^+^ T-cell counts (*P* = 0.041; Supplementary Table S2), suggesting a differential longitudinal trajectory that warrants further investigation. No significant changes were observed in CD8^+^ T-cell activation markers CD38 and HLA-DR (Supplementary Table S2).

### Inflammatory cytokine parameters

**FIGURE 3:**
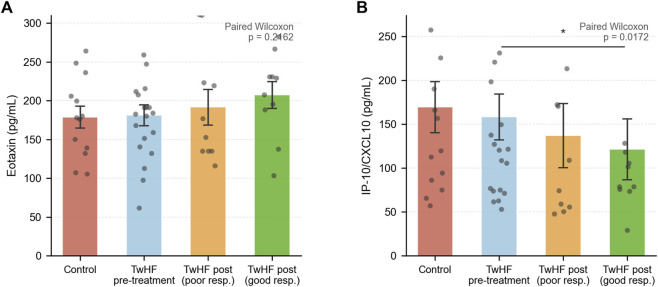
Eotaxin and IP-10/CXCL10 levels in control participants and TwHF-treated individuals. **(A)** Plasma eotaxin and **(B)** plasma IP-10/CXCL10 concentrations in control participants (n = 14) and TwHF-treated individuals before treatment (n = 20) and after treatment, stratified by immunological response (poor responders: lower half by CD4^+^ gain, n = 10; good responders: upper half, n = 10). Bars = group mean ± SEM; individual data points overlaid. Paired Wilcoxon signed-rank test for pre- vs. post-treatment comparison within the TwHF cohort. *P < 0.05.

Cross-sectional comparisons among controls, TwHF pre-treatment samples, and post-treatment samples stratified by immunological response did not reveal significant between-group differences for IP-10/CXCL10 or eotaxin (Figure 3). However, paired analyses within the TwHF cohort demonstrated a significant within-person reduction in IP-10/CXCL10 after treatment (median Δ −31.96 pg/mL, *P* = 0.017; Figure 3), which was more pronounced in good responders. Eotaxin levels showed a positive numerical trend (median Δ +13.11 pg/mL) that did not reach statistical significance (*P* = 0.216; Figure 3A).

### Immunological response and subgroup analyses

A clinically meaningful immunological response (≥50 cells/μL/year) was observed in 75.0% of TwHF-treated individuals versus 9.7% of controls. Response rates were comparable between participants with baseline CD4^+^ T-cell counts <200 and ≥200 cells/μL within the TwHF cohort.

## Discussion

### TwHF accelerates CD4^+^ T-cell reconstitution in INR

A central finding of this study is that TwHF was associated with a significantly faster rate of CD4^+^ T-cell recovery compared with matched controls. Longitudinal mixed-effects modeling revealed a significant group × time interaction, indicating that the immune benefit was temporally linked to TwHF exposure. This effect was most pronounced in the second half of the intervention, suggesting that sustained immunomodulation may be required before clinically meaningful CD4^+^ gains are observed.

The advantage conferred by TwHF was not maintained after treatment withdrawal, as CD4^+^ T-cell trajectories converged during post-intervention follow-up. This reversible pattern implies that TwHF exerts a treatment-dependent modulatory effect rather than permanently resetting immune homeostasis (Li et al., 2025). These findings are consistent with, and extend, the evidence base established by our group’s prior work. The multi-omics study by Liu et al. identified suppression of T-cell activation pathways as a key mechanism of TwHF in INR patients (Liu et al., 2022), providing mechanistic context for the immunological effects documented here. Notably, the randomized phase II trial of LLDT-8—a synthetic triptolide analogue that is chemically distinct from the TwHF botanical preparation evaluated in the present study—similarly demonstrated CD4^+^ T-cell gains in virologically suppressed PLWH with INR (Cao et al., 2023), suggesting that triptolide-related mechanisms may underlie the immunological effects of both agents despite their pharmacological differences. The present study advances this body of evidence by characterizing the subset-level dynamics and temporal trajectory of immune recovery—dimensions not addressed in prior investigations—and by documenting the reversibility of the immunological effect following treatment discontinuation.

### Preferential expansion of memory but not naïve CD4^+^ T cells

The selective expansion of memory CD4^+^ T cells, with no corresponding change in naïve counts, reflects the immunological reality of the INR population studied. In PLWH with long-standing HIV infection and prolonged immunodeficiency, thymic output is substantially diminished, limiting the capacity for *de novo* naïve T-cell generation regardless of immunomodulatory intervention (Dos Santos Guedes et al., 2023). Consequently, peripheral memory CD4^+^ T-cell dynamics become the dominant determinant of net CD4^+^ recovery in this context.

A key pathological feature of INR is the coexistence of elevated CD4^+^ T-cell proliferation and reduced net CD4^+^ counts—a paradox explained by increased activation-driven apoptosis predominantly affecting the memory subset. Zhang et al. demonstrated that Ki67^+^CD4^+^ T cells are enriched in INRs and exhibit impaired survival capacity relative to naïve CD4^+^ T cells, indicating that pathological proliferation accelerates memory CD4^+^ turnover without yielding net expansion (Zhang et al., 2024). Compounding this, Xiao et al. showed that even in virally suppressed PLWH, stem cell-like memory T cells (TSCM) retain residual exhaustion signatures—including reduced TCF-1 expression and elevated TOX—that impair their self-renewal potential (Xiao et al., 2025). Within this framework, TwHF’s suppression of IFN-γ–driven inflammatory signaling may reduce the activation stimulus that drives memory CD4^+^ T cells into this high-turnover, apoptosis-prone state, thereby permitting net memory subset accumulation. This interpretation is consistent with the observation that the TwHF benefit was most pronounced in the second half of the treatment period, suggesting that sustained attenuation of inflammatory pressure is required before memory pool recovery becomes detectable. Whether TwHF directly modulates apoptotic or survival signaling in CD4^+^ T-cell subpopulations requires dedicated mechanistic investigation.

### Effects on CD4/CD8 ratio and CD8^+^ T-cell dynamics

TwHF treatment was associated with a more rapid improvement in the CD4/CD8 ratio (group × time *P* = 0.001), a clinically relevant marker of immune aging and non-AIDS comorbidity risk (Ron et al., 2023; Martínez-Sanz et al., 2023; Serrano-Villar et al., 2022; Ron et al., 2024). The pre-treatment difference in CD8^+^ T-cell counts between groups (lower in TwHF at Pre-M12 and TwHF-M0) is noteworthy. Although pre-ART baseline CD8^+^ counts were comparable, a between-group discrepancy emerged at the longitudinal baseline. This likely reflects differences in cohort entry timing relative to the natural course of CD8^+^ T-cell dynamics during long-term ART, a limitation acknowledged below. Importantly, this imbalance resolved by TwHF-M6, and no significant CD8^+^ T-cell count differences were observed during the treatment period at cross-sectional time points. The nominally significant group × time interaction for CD8^+^ T-cell counts in the linear mixed-effects model (*P* = 0.041) reflects the convergence of initially divergent trajectories rather than a distinct immunological effect of TwHF on the CD8^+^ compartment.

In contrast to the CD4^+^ compartment, no significant changes were observed in CD8^+^ T-cell activation markers including CD38 and HLA-DR expression. This selective immunological profile is consistent with a mechanism of immune recalibration rather than generalized immunosuppression.

### IP-10 reduction and inflammatory modulation

The within-cohort reduction in IP-10/CXCL10 after TwHF treatment (median Δ −31.96 pg/mL, *P* = 0.017) provides mechanistic grounding for the observed CD4^+^ T-cell recovery. IP-10/CXCL10 is a downstream effector of IFN-γ signaling and a consistent correlate of chronic immune activation in ART-treated PLWH; its plasma levels correlate inversely with CD4^+^ T-cell counts and directly with CD8^+^ T-cell activation markers over the course of long-term ART (Rajasuriar et al., 2010; Kroeze et al., 2021; Ploquin et al., 2016; Wan et al., 2023; Zhang et al., 2023). Triptolide, the principal bioactive component of TwHF, suppresses CXCL10 transcription *in vitro* through inhibition of the JAK/STAT1 axis downstream of IFN-γ receptor signaling (Shi et al., 2023), providing a plausible molecular basis for the within-patient IP-10 decline observed here. The preferential reduction in good immunological responders is consistent with a mechanistic link between IP-10 suppression and CD4^+^ T-cell recovery, though the directionality of this relationship—whether IP-10 reduction precedes or follows immune improvement—cannot be established from the present cross-sectional cytokine design.

Eotaxin levels showed a non-significant numerical increase after TwHF treatment (median Δ +13.11 pg/mL, *P* = 0.216). Given the small cytokine substudy sample and the absence of statistical significance, this finding is considered exploratory and is not further interpreted.

### Immunological responders and clinical implications

The response rate in the TwHF group (75.0%) was markedly higher than in controls (9.7%), underscoring the robustness of the treatment effect at the individual level. The lack of a significant difference between CD4^+^ <200 and ≥200 cells/μL subgroups within the TwHF cohort suggests that TwHF benefit may be relatively independent of the degree of immunodeficiency at treatment initiation, supporting its potential broad applicability across the INR spectrum.

### Study limitations

Several limitations warrant acknowledgement. First, the retrospective single-center design and modest sample size may introduce selection bias and limit generalizability. Second, the pre-treatment discrepancy in CD8^+^ T-cell counts between groups (higher in controls at Pre-M12 and TwHF-M0) was not present at the pre-ART baseline and likely reflects differences in cohort entry timing relative to the natural CD8^+^ T-cell decline trajectory during long-term viral suppression. Although this imbalance resolved during the treatment period and is unlikely to confound the primary CD4^+^ T-cell recovery outcomes, it represents a source of non-comparability that cannot be fully excluded. Third, the cytokine substudy comprised a limited sample (n = 20 TwHF participants, n = 14 controls), which may have restricted statistical power, particularly for eotaxin analyses. Fourth, the immunophenotyping panel employed in this study assessed CD8^+^ T-cell activation (CD38^+^ and HLA-DR^+^) but did not include activation, senescence (CD57), or exhaustion (PD-1) markers on CD4^+^ T cells. Given emerging evidence that residual exhaustion of memory CD4^+^ T-cell subpopulations—including TSCM—persists in virally suppressed PLWH (Xiao et al., 2025), and that pathological proliferation with impaired survival characterizes the memory CD4^+^ compartment in INR (Zhang et al., 2024), future studies incorporating these markers would provide a more comprehensive characterization of the immunological mechanisms underlying TwHF-associated CD4^+^ recovery. Finally, more detailed mechanistic studies incorporating tissue-level immune profiling and functional assays are warranted.

## Conclusion

This study demonstrates that TwHF significantly accelerates CD4^+^ T-cell recovery in ART-treated PLWH with INR, primarily through expansion of memory CD4^+^ T cells and improvement of the CD4/CD8 ratio. A significant within-cohort reduction in IP-10/CXCL10 supports modulation of interferon-γ–associated inflammatory signaling as a contributing mechanism. The immunological benefit is reversible and treatment-dependent, consistent with a modulatory rather than regenerative mechanism. These findings extend the evidence base established by prior prospective work from our group and support TwHF as a candidate adjunctive immunomodulatory therapy for INR, providing a rationale for prospective, multicenter studies with mechanistic and clinical endpoint integration.

## Data Availability

The datasets analyzed during the current study are available from the first author on reasonable request.
