# Supplementary figures and images for: Tripterygium wilfordii Hook F accelerates CD4+ T-cell recovery in ART-treated people living with HIV with incomplete immune reconstitution: a longitudinal cohort study
Source: Front Pharmacol. 2026 Jun 12;17:1854636. doi: 10.3389/fphar.2026.1854636 (PMC13299095; doi:10.3389/fphar.2026.1854636)

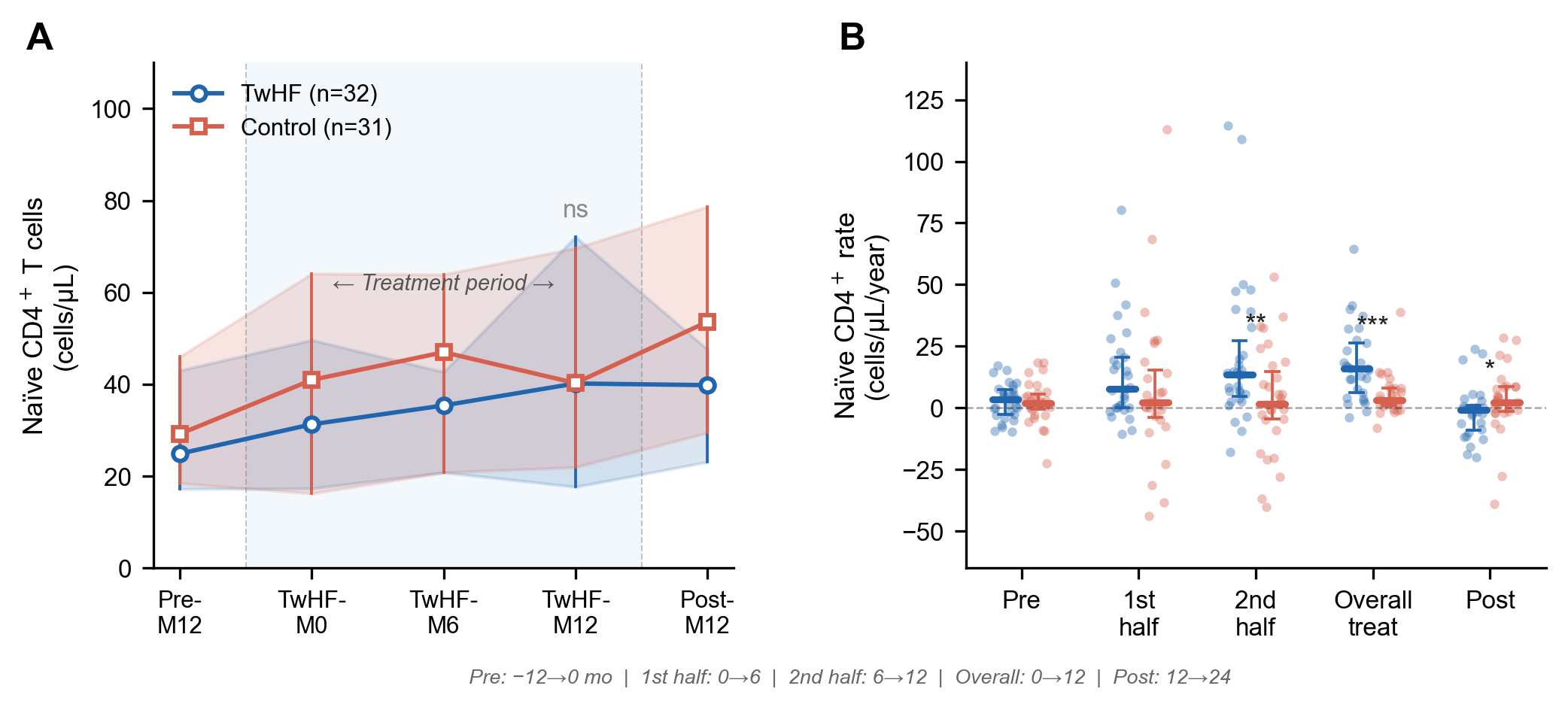

Supplement: Supplementary file 1 [file Image1.tiff]

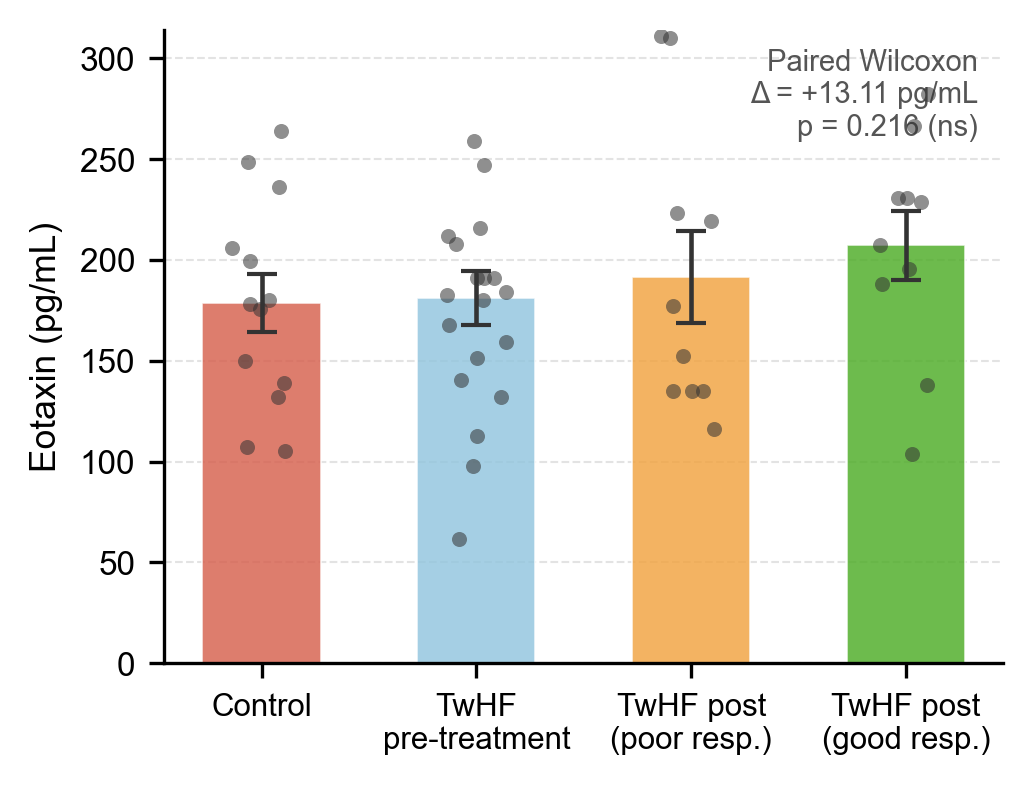

Supplement: Supplementary file 3 [file Image2.tiff]
