# Supplementary material for: Tripterygium wilfordii Hook F accelerates CD4+ T-cell recovery in ART-treated people living with HIV with incomplete immune reconstitution: a longitudinal cohort study
Source: Front Pharmacol. 2026 Jun 12;17:1854636. doi: 10.3389/fphar.2026.1854636 (PMC13299095; doi:10.3389/fphar.2026.1854636)
Supplement: Supplementary file 2 [file Supplementaryfile1.docx]

**Supplementary Material**

*Tripterygium wilfordii Hook F accelerates CD4⁺ T-cell recovery in ART-treated people living with HIV with incomplete immune reconstitution: a longitudinal cohort study*

**Supplementary Figure Legends**

**Supplementary Figure S1. Naïve CD4⁺ T-cell longitudinal dynamics.**

(A) Absolute counts of naïve CD4⁺ T cells at five predefined time points (Pre-M12, TwHF-M0, TwHF-M6, TwHF-M12, Post-M12). Data are medians with IQR (shaded area); the treatment period is highlighted. No significant between-group differences were observed at any time point (all *P*>0.05, Mann–Whitney U test), confirming that CD4⁺ T-cell recovery in the TwHF group was attributable to expansion of memory rather than naïve CD4⁺ T-cell subsets. (B) Rates of change in naïve CD4⁺ T-cell counts (cells/μL/year, annualised) during the pre-treatment, treatment, and post-treatment periods. Horizontal lines = group medians; vertical bars = IQR; individual data points overlaid. ***P*<0.01, ****P*<0.001 by Mann–Whitney U test. Asterisks in panel B denote significant between-group differences in annualised rate of change (TwHF vs. control) within each interval, by Mann–Whitney U test; they do not reflect absolute count differences at individual time points. Rates for 6-month intervals (0→6 mo and 6→12 mo) were annualised by multiplying by 2.

**Supplementary Figure S2. Eotaxin levels following TwHF treatment.**

Plasma eotaxin concentrations in control participants (n=14) and TwHF-treated individuals before treatment (n=20) and after treatment, stratified by immunological response (poor responders: lower half by CD4⁺ T-cell gain, n=10; good responders: upper half, n=10). Bars = group mean ± SEM; individual data points are overlaid. Paired Wilcoxon signed-rank test for pre- vs. post-treatment comparison within the TwHF cohort yielded median Δ = +13.11 pg/mL (*P*=0.216, not significant). The numerical trend toward higher eotaxin levels in good responders did not reach statistical significance and is considered an exploratory observation.

**Supplementary Table S1. CD8⁺ T-cell counts across follow-up time points.**

Absolute CD8⁺ T-cell counts (cells/μL) at each predefined time point. Between-group comparisons by Mann–Whitney U test. The pre-ART baseline (t0) was comparable between groups. The discrepancy observed at Pre-M12 and TwHF-M0 likely reflects differences in the timing of cohort entry relative to the natural CD8⁺ T-cell trajectory during long-term viral suppression. This difference resolved by TwHF-M6 and was not significant at subsequent time points. **P*<0.05, ***P*<0.01.

| **Timepoint** | **TwHF median [IQR]** | **n** | **Control median [IQR]** | **n** | **P value** | **Note** |
| --- | --- | --- | --- | --- | --- | --- |
| Pre-ART (t0) | 540 [411–788] | 32 | 627 [394–927] | 31 | 0.611 | ART-initiation baseline; groups comparable |
| Pre-M12 | 550 [440–814] | 32 | 839 [604–1011] | 31 | **0.005**** | Pre-treatment difference; converges during treatment |
| TwHF-M0 | 626 [490–737] | 32 | 739 [585–1012] | 31 | **0.044*** | Pre-treatment difference; converges during treatment |
| TwHF-M6 | 624 [439–827] | 32 | 654 [520–860] | 31 | 0.616 |  |
| TwHF-M12 | 714 [539–850] | 32 | 747 [547–967] | 31 | 0.505 |  |
| Post-M12 | 636 [494–788] | 28 | 709 [492–870] | 31 | 0.490 |  |

**Supplementary Table S2. Linear mixed-effects model results — treatment period.**

Group × time interaction coefficients from linear mixed-effects models for each immunological parameter during the treatment period (TwHF-M0, TwHF-M6, TwHF-M12). A significant positive interaction coefficient indicates a faster rate of change in the TwHF group relative to controls. Time = months from TwHF-M0 (0, 6, 12). Group coded as 1 (TwHF) or 0 (control). CD4⁺ T-cell counts also modeled across the full follow-up (all five time points). SE = standard error.

| **Parameter** | **Coef_time** | **P_time** | **Coef_interaction** | **P_interaction** | **N_obs** |
| --- | --- | --- | --- | --- | --- |
| Total CD4⁺ T cells — treatment period (cells/μL) | 1.852 | 0.007 | 4.98 | **<0.001** | 189 |
| Total CD4⁺ T cells — full follow-up (cells/μL) | 1.465 | <0.001 | 1.439 | **<0.001** | 311 |
| Memory CD4⁺ T cells — treatment period (cells/μL) | 1.252 | 0.028 | 4.303 | **<0.001** | 181 |
| Naïve CD4⁺ T cells — treatment period (cells/μL) | 0.808 | 0.030 | 0.326 | 0.529 | 180 |
| CD4/CD8 ratio — treatment period | 0.001 | 0.455 | 0.007 | **0.001** | 186 |
| CD8⁺ T cells — treatment period (cells/μL) | −5.140 | 0.145 | 10.080 | **0.041** | 189 |
| CD8⁺CD38⁺ (%) — treatment period | −0.114 | 0.664 | 0.361 | 0.328 | 181 |
| CD8⁺HLA-DR⁺ (%) — treatment period | 0.249 | 0.183 | 0.042 | 0.873 | 181 |

**Supplementary Table S3. Naïve and memory CD4⁺ T-cell absolute counts at each time point.**

Absolute counts (cells/μL) computed as CD4⁺ T-cell count × percentage of naïve (CD45RA⁺CD62L⁺) or memory (CD45RA⁻) subsets. Between-group comparisons by Mann–Whitney U test.

| **Timepoint** | **Subset** | **TwHF median [IQR]** | **n** | **Control median [IQR]** | **n** | **P value** |
| --- | --- | --- | --- | --- | --- | --- |
| Pre-M12 | Memory | 120 [106–175] | 29 | 136 [119–164] | 31 | 0.584 |
| Pre-M12 | Naïve | 25 [17–43] | 29 | 29 [18–46] | 31 | 0.564 |
| TwHF-M0 | Memory | 146 [98–175] | 29 | 143 [129–184] | 29 | 0.576 |
| TwHF-M0 | Naïve | 31 [17–50] | 29 | 41 [16–64] | 28 | 0.528 |
| TwHF-M6 | Memory | 167 [134–201] | 31 | 156 [131–194] | 31 | 0.464 |
| TwHF-M6 | Naïve | 35 [21–43] | 31 | 47 [21–64] | 31 | 0.311 |
| TwHF-M12 | Memory | 216 [186–243] | 32 | 164 [132–198] | 29 | **<0.001** |
| TwHF-M12 | Naïve | 40 [18–72] | 32 | 40 [22–70] | 29 | 0.723 |
| Post-M12 | Memory | 207 [161–254] | 26 | 174 [143–211] | 26 | 0.069 |
| Post-M12 | Naïve | 40 [23–48] | 26 | 54 [29–79] | 26 | 0.224 |

**Supplementary Table S4. CD4/CD8 ratio at each follow-up time point.**

Between-group comparisons by Mann–Whitney U test. The Pre-M12 difference reflects the corresponding CD8⁺ T-cell baseline imbalance (see Supplementary Table S1 and Limitations section). **P*<0.05, ***P*<0.01.

| **Timepoint** | **TwHF median [IQR]** | **n** | **Control median [IQR]** | **n** | **P value** |
| --- | --- | --- | --- | --- | --- |
| Pre-M12 | 0.277 [0.194–0.441] | 32 | 0.211 [0.154–0.292] | 31 | **0.026*** |
| TwHF-M0 | 0.287 [0.220–0.413] | 31 | 0.271 [0.143–0.354] | 31 | 0.254 |
| TwHF-M6 | 0.304 [0.253–0.445] | 32 | 0.279 [0.225–0.409] | 31 | 0.353 |
| TwHF-M12 | 0.409 [0.312–0.480] | 30 | 0.278 [0.214–0.361] | 31 | **0.008**** |
| Post-M12 | 0.382 [0.302–0.504] | 27 | 0.305 [0.242–0.436] | 31 | 0.115 |

**Supplementary Table S5. Sensitivity analysis: CD4⁺ T-cell recovery model adjusted for baseline CD8⁺ T-cell counts.**

Linear mixed-effects models for total CD4⁺ T-cell counts during the treatment period (TwHF-M0, TwHF-M6, TwHF-M12), with and without adjustment for pre-treatment CD8⁺ T-cell counts. Sensitivity models address the pre-treatment CD8⁺ T-cell imbalance between groups (see Supplementary Table S1). The stability of the group × time interaction across all models confirms that the CD8⁺ baseline discrepancy does not confound the primary immunological outcome. Coef = coefficient; SE = standard error.

| **Model** | **Covariate** | **Coef_time** | **Coef_interaction** | **P_interaction** | **N_obs** | **N_subjects** |
| --- | --- | --- | --- | --- | --- | --- |
| Primary model (unadjusted) | None | 1.852 | 4.98 | **<0.001** | 189 | 63 |
| Sensitivity 1 | CD8⁺ at TwHF-M0 (P=0.429) | 1.852 | 4.98 | **<0.001** | 189 | 63 |
| Sensitivity 2 | CD8⁺ at Pre-M12 (P=0.627) | 1.852 | 4.98 | **<0.001** | 189 | 63 |

CD8⁺ covariate P-values shown in parentheses. Coef_interaction = group × time interaction coefficient, identical across all three models (4.98), indicating that adjustment for pre-treatment CD8⁺ T-cell counts does not alter the estimated rate advantage of TwHF treatment.

**Supplementary Table S6. Routine safety parameters across follow-up time points.**

Hematological and biochemical safety parameters at each predefined time point. Values are group medians [IQR]. Between-group comparisons by Mann–Whitney U test. Timepoints correspond to clinical laboratory assessments matched to the nearest available measurement within a ±3-month window of each study visit. All values were within clinically acceptable limits throughout the observation period.

**WBC (×10⁹/L)**

| **Timepoint** | **TwHF median [IQR]** | **n** | **Control median [IQR]** | **n** | **Normal range** | **P value** |
| --- | --- | --- | --- | --- | --- | --- |
| Pre-M12 | 4.7 [4.1–5.6] | 26 | 5.1 [4.2–6.6] | 20 | 3.5–9.5 | 0.369 |
| TwHF-M0 | 5.1 [3.7–6.1] | 30 | 5.1 [4.0–6.2] | 24 | 3.5–9.5 | 0.583 |
| TwHF-M6 | 5.1 [3.4–6.0] | 28 | 5.2 [4.1–5.6] | 24 | 3.5–9.5 | 0.890 |
| TwHF-M12 | 5.0 [4.1–6.3] | 28 | 5.9 [4.8–6.7] | 23 | 3.5–9.5 | 0.394 |
| Post-M12 | 5.7 [4.5–6.3] | 25 | 5.5 [4.5–6.0] | 15 | 3.5–9.5 | 0.576 |

**Hemoglobin (g/L)**

| **Timepoint** | **TwHF median [IQR]** | **n** | **Control median [IQR]** | **n** | **Normal range** | **P value** |
| --- | --- | --- | --- | --- | --- | --- |
| Pre-M12 | 147.0 [140.2–157.0] | 26 | 140.0 [134.8–149.2] | 20 | 120–160 | 0.169 |
| TwHF-M0 | 148.5 [135.2–156.5] | 30 | 143.5 [136.5–151.0] | 24 | 120–160 | 0.361 |
| TwHF-M6 | 147.0 [142.0–155.0] | 29 | 147.5 [140.0–163.2] | 24 | 120–160 | 0.537 |
| TwHF-M12 | 148.0 [135.8–152.8] | 28 | 149.0 [141.0–155.5] | 23 | 120–160 | 0.622 |
| Post-M12 | 153.0 [145.0–156.0] | 25 | 147.0 [135.5–159.0] | 15 | 120–160 | 0.605 |

**Platelet count (×10³/μL)**

| **Timepoint** | **TwHF median [IQR]** | **n** | **Control median [IQR]** | **n** | **Normal range** | **P value** |
| --- | --- | --- | --- | --- | --- | --- |
| Pre-M12 | 212.0 [178.2–240.5] | 26 | 212.0 [197.5–257.8] | 20 | 100–300 | 0.506 |
| TwHF-M0 | 208.0 [179.2–229.5] | 30 | 229.5 [188.5–262.8] | 24 | 100–300 | 0.169 |
| TwHF-M6 | 206.0 [179.8–233.0] | 28 | 219.5 [192.8–260.5] | 24 | 100–300 | 0.321 |
| TwHF-M12 | 210.5 [163.5–256.2] | 28 | 218.0 [202.0–232.0] | 23 | 100–300 | 0.538 |
| Post-M12 | 199.0 [165.0–244.0] | 25 | 210.0 [194.5–230.5] | 15 | 100–300 | 0.539 |

**Serum creatinine (μmol/L)**

| **Timepoint** | **TwHF median [IQR]** | **n** | **Control median [IQR]** | **n** | **Normal range** | **P value** |
| --- | --- | --- | --- | --- | --- | --- |
| Pre-M12 | 77.0 [67.5–83.0] | 27 | 74.5 [68.0–82.0] | 18 | 44–133 | 0.954 |
| TwHF-M0 | 76.0 [67.5–79.5] | 27 | 75.0 [69.2–84.2] | 18 | 44–133 | 0.763 |
| TwHF-M6 | 73.0 [68.8–77.8] | 28 | 78.5 [69.2–86.2] | 22 | 44–133 | 0.240 |
| TwHF-M12 | 74.0 [65.8–80.5] | 28 | 75.5 [68.0–85.0] | 22 | 44–133 | 0.604 |
| Post-M12 | 73.5 [69.8–77.2] | 24 | 70.7 [63.5–77.0] | 15 | 44–133 | 0.497 |

**Liver transaminases (U/L)**

| **Timepoint** | **Enzyme** | **TwHF median [IQR]** | **n** | **Control median [IQR]** | **n** | **ULN** |  | **P value** |
| --- | --- | --- | --- | --- | --- | --- | --- | --- |
| Pre-M12 | ALT | 24.0 [17.0–38.0] | 27 | 23.0 [17.5–29.0] | 19 | <40 |  | 0.729 |
| Pre-M12 | AST | 23.0 [19.5–31.5] | 26 | 22.0 [18.8–27.5] | 12 | <40 |  | 0.625 |
| TwHF-M0 | ALT | 28.0 [24.0–37.0] | 29 | 28.5 [21.0–39.8] | 18 | <40 |  | 0.861 |
| TwHF-M0 | AST | 25.0 [21.0–30.0] | 25 | 25.0 [21.0–33.0] | 15 | <40 |  | 0.856 |
| TwHF-M6 | ALT | 29.0 [21.0–44.0] | 29 | 28.0 [17.0–34.0] | 23 | <40 |  | 0.555 |
| TwHF-M6 | AST | 26.5 [20.2–32.2] | 26 | 23.0 [20.5–30.5] | 19 | <40 |  | 0.629 |
| TwHF-M12 | ALT | 34.5 [23.0–55.0] | 28 | 26.0 [20.8–34.5] | 22 | <40 |  | 0.190 |
| TwHF-M12 | AST | 28.0 [24.0–36.0] | 27 | 24.5 [20.2–33.0] | 18 | <40 |  | 0.301 |
| Post-M12 | ALT | 35.0 [23.5–49.5] | 24 | 27.0 [18.0–41.0] | 15 | <40 |  | 0.516 |
| Post-M12 | AST | 28.0 [21.0–31.5] | 23 | 22.0 [21.0–28.0] | 11 | <40 |  | 0.407 |

ULN = upper limit of normal. The ≤2×ULN elevations in liver transaminases reported in the main text (8 TwHF participants, 3 controls) were asymptomatic and self-resolving; median ALT and AST values remained below the ULN threshold at all time points in both groups. Between-group differences at all time points: all *P*>0.05.
